# Glucagon-Like Peptide-1 Agonist Exendin-4 Facilitates the Extinction of Cocaine-Induced Condition Place Preference

**DOI:** 10.3389/fnsys.2021.711750

**Published:** 2021-11-24

**Authors:** Changliang Zhu, Tao Hong, Hailiang Li, Shucai Jiang, Baorui Guo, Lei Wang, Jiangwei Ding, Caibin Gao, Yu Sun, Tao Sun, Feng Wang, Yangyang Wang, Din Wan

**Affiliations:** ^1^Department of Neurosurgery, General Hospital of Ningxia Medical University, Yinchuan, China; ^2^Ningxia Key Laboratory of Cerebrocranial Disease, Incubation Base of National Key Laboratory, Ningxia Medical University, Yinchuan, China; ^3^Department of Neurosurgery, The First Affiliated Hospital of Nanchang University, Nanchang, China; ^4^Department of Neurosurgery, The First Affiliated Hospital of Zhejiang University School of Medicine, Hangzhou, China

**Keywords:** cocaine, condition place preference, extinction, reinstatement, exendin-4, toll-like receptor 4

## Abstract

Accumulating studies suggest that the glucagon-like peptide-1 receptor agonist exendin-4 (Ex4) and toll-like receptor 4 (TLR4) play a pivotal role in the maladaptive behavior of cocaine. However, few studies have assessed whether Ex4 can facilitate the extinction of drug-associated behavior and attenuate the reinstatement of cocaine-induced condition place preference (CPP) in mice. The main objective of the present study was to evaluate Ex4’s ability to regulate the extinction and reinstatement of cocaine-induced CPP. C57BL/6 mice were conditioned to either cocaine (20 mg/kg) or an equivalent volume of saline to establish a cocaine-mediated CPP paradigm. To investigate the potential effects of Ex4 on extinction, animals received an intraperitoneal injection of Ex4 either immediately or 6 h after each extinction or only on the test day. The persistence of extinction was measured using the reinstatement paradigm evoked by 10 mg/kg of cocaine. To explore the possible impacts of Ex4 and neuroinflammation on cocaine, the expression levels of TLR4 within the hippocampus was detected using western blotting. As a result, we found that systemic administration of Ex4 immediately after each extinction training, instead of 6 h after each extinction and on the day of extinction test, was capable of facilitating extinction in the confined or non-confined CPP extinction paradigms and blocking the cocaine-primed reinstatement of cocaine-induced CPP. Additionally, we also observed that Ex4 was competent to alleviate TLR4 signaling that has been up-regulated by cocaine. Altogether, our findings indicated that the combination of Ex4 with daily extinction training was sufficient to facilitate extinction of the conditioned behavior, attenuate reinstatement of cocaine-induced CPP and inhibit TLR4 signaling. Thus, Ex4 deserves further investigation as a potential intervention for the treatment of cocaine use disorder.

## Introduction

Cocaine addiction is a chronic brain disorder characterized by a high proportion of relapse to compulsive behavior of cocaine intake even following a prolonged period of detoxification (Koob and Volkow, 2016). The development of clinical treatments for substance abuse was greatly hampered by relapse (O’Brien, 1997; Vorel et al., 2001), which can be precipitated by re-exposure to environmental cues involved in the rewarding properties of drugs and re-exposure to the drug itself (O’Brien et al., 1992; Torregrossa and Taylor, 2013; Koob and Volkow, 2016). However, the efficacy of the available medications for the treatment of cocaine use disorder appears to be limited to date (O’Brien, 1997). Hence, additional research is essential to treat cocaine abuse and reduce relapse (Jerlhag, 2019). Interestingly, in the search for novel agents to treat cocaine addiction, previous work found that medications which can impair the ability of cocaine-context cues to trigger craving by reducing the strength of memories formed between the rewarding effects of cocaine and environment have been shown to be effective at reducing relapse after re-exposure to cocaine-associated stimuli (Torregrossa and Taylor, 2013).

Memory, which is strongly associated with relapse-like behavior (i.e., reinstatement; Lee et al., 2006), is a time-dependent mental process mainly composed of three phases: encoding, retaining, and retrieving information. Once this memory is established and retrieved, it enters a short-term destabilization period which either fades or is encoded and undergoes an active long-term storage process. In general, extinction training is considered as an active process that results in a progressive decline in acquired reactions. It produces the normal formation of learning and memories that predicts no more delivery of addictive drugs with rewarding and pleasurable properties, thereby competing against the expression of original drug-related memories to control behavior (Marlatt, 1990; Millan et al., 2011; McNally, 2014; Chesworth and Corbit, 2015). Furthermore, preclinical studies in animal models also support that repeatedly exposing subjects to previous drug-related compartments in the absence of drugs helps to increase the period of abstinence (Havermans and Jansen, 2003; Kelamangalath et al., 2007; Xue et al., 2012). In addition, it has been demonstrated that extinction can lead to the suppression of the original drug-related memories (Marlatt, 1990; Millan et al., 2011; McNally, 2014), thereby reducing the risk of relapse (Heather and Bradley, 1990). Taken together, these data support that weakening the drug-context memories using extinction training effectively reduces the susceptibility of relapse triggered by drug-context memories (Torregrossa and Taylor, 2016).

Intriguingly, neuroinflammation has attracted increasing interest due to the profound influence on neural adaptations following chronic drugs exposure (Pocock and Kettenmann, 2007; Lacagnina et al., 2017). In addition, memory processes are thought to be closely related to neuroinflammation (Bader and Winklhofer, 2020; Correia et al., 2020), which is triggered by the activation of pattern recognition receptors (PRRs), including toll-like receptors 4 (TLR4) primarily expressed on microglia (Hanamsagar et al., 2012). TLR4 could recognize cocaine as foreign danger-associated compound and then initiate central innate immune in response to the potential threats (Northcutt et al., 2015). The over-expression of TLR4 has also been shown to attenuate drug reward learning and behavior (Kashima and Grueter, 2017). In addition, blocking neuroinflammatory reaction to cocaine by anti-inflammatory agents, both in rodents and humans, has shown efficacy in the treatment of substance abuse by acting on drug intake, craving, or relapse (Ray et al., 2014; Kohno et al., 2019). For instance, pharmacological antagonism of TLR4 has also been demonstrated to attenuate cocaine-primed reinstatement (Brown et al., 2018). Collectively, these data suggested that anti-inflammatory agents that block TLR4 activation may be beneficial for enhancing extinction of cocaine-associated behavior and reducing cocaine-primed reinstatement. Indeed, extensive studies have also further confirmed that pharmacological interventions are effective at enhancing the extinction of substance abuse (Botreau et al., 2006; Paolone et al., 2009; Chesworth and Corbit, 2015), and thereby decreasing the risk of relapse. However, few literatures have reported the effect of anti-inflammatory agents in cocaine addiction to date (Correia et al., 2020).

Exendin-4 (Ex4) is a glucagon-like peptide-1 (GLP-1) receptor (GLP-1R) agonist that possesses a long half-life and can cross the blood-brain barrier (Andersen et al., 2018). GLP-1 is an anorexigenic peptide released by intestinal L cells and preproglucagon neurons, located in the nucleus tractus solitarius of the caudal brainstem (Holst, 2007; Grill and Hayes, 2012). GLP-1 binds to GLP-1R, a widely expressed receptor in hippocampal CA3 pyramidal neurons (Lee et al., 2018), which is an essential region for learning and memory (Kim and Fanselow, 1992; Bohbot and Corkin, 2007). Moreover, Ex4 was considered as a novel anti-inflammatory agent due to its suppressive effects on the mRNA expression of many indices of inflammation including TLR-2, TLR-4, TNF-α, and IL-1β (Ajay et al., 2011) and improve behavioral performance, such as learning and memory (Gault and Hölscher, 2018; Mandal et al., 2018) which plays a pivotal role in drug addiction (Hyman et al., 2006). Nonetheless, to the best of our knowledge, few studies have investigated whether the anti-inflammatory agent Ex4 accompanied by extinction training would produce the new formations of learning during extinction which reversely compete against the original memories, thereby contributing to promote extinction and attenuate the reinstatement of cocaine-induced CPP in mice.

To answer these critical questions mentioned above, we sought to investigate the effects of the novel anti-inflammatory agent Ex4 on the extinction and reinstatement of cocaine-induced CPP. Additionally, we also explored whether these behavioral actions of Ex4 are associated with the time of injection known as a time window of extinction memory consolidation. Furthermore, we measured the changes of TLR4 expression in the hippocampus of the cocaine-experienced mice.

## Materials and Methods

### Animals

All adult male C57BL/6J mice (18–22 g weight) used in this study were purchased from the Experimental Animal Center of Ningxia Medical University (China). Four animals were maintained per cage with a 12-h light/dark cycle with illumination on at 07:00 AM and lights off at 07:00 PM. The ambient temperature and humidity of the vivarium were set at 20–25°C and 50%–60%. Mice had access to standard rodent chow and tap water *ad libitum*. Mice were habituated for one weight, and their weight was controlled daily (20–25 g). After transferring from the colony room, all mice were acclimated to handling in the place-conditioning room for 30 min on consecutive days prior to the start of the behavioral experimentation. Intraperitoneal injections were alternately conducted on the left or right abdomen. In addition, animals were acclimated to contact with other animals before the commencement of the experiments. All efforts were made to reduce the psychological stress of animals caused by the novel environment and repeated intraperitoneal injections. The animal study was approved by the Ningxia Key Laboratory of Cerebrocranial Disease, and all experimental procedures related to mice were approved by the national, regional, and local laws and regulations and the guidelines established by the Animal Research Ethics Committee of Ningxia Medical University. The primary purpose of our efforts was to reduce animal suffering and minimize the number of mice used in our research.

### Drugs and Antibodies

Cocaine-hydrochloride (HCl) was purchased from China National Medicines Corporation Limited (Beijing, China). Cocaine-HCl was dissolved in 0.9% physiological saline solution (0.9% NaCl) and was injected at a concentration of 4 mg/ml on alternate days. The cocaine-induced CPP paradigm was performed to examine cocaine CPP extinction and reinstatement. The dose for conditioning (20 mg/kg) that elicits a strong CPP behavior was chosen based on previous studies (Meye et al., 2016; Ladrón de Guevara-Miranda et al., 2019). Cocaine was prepared for intraperitoneal (i.p.) administration immediately before use at doses of 20 mg/kg body weight to induce the CPP paradigm or 10 mg/kg body weight to induce CPP reinstatement. Saline was used as a vehicle solution. Ex4 was freshly prepared at 1.0 μg/kg body weight, which reportedly decreases the locomotor activating effects of cocaine (Sorensen et al., 2015). The GLP-1R agonist Exendin-4 (Ex4) was purchased from MedChemExpress (MCE, USA) and was diluted in 0.9% physiological saline solution at a concentration of 0.02 μg/ml accordingly. Injections of Ex4 and cocaine-HCl were carried out alternatively on the left or right side of the peritoneum. Ex4 was administered 0 h and 6 h after the extinction procedure or as a single injection on the extinction test day. TLR4 antibody was purchased from Abcam (San Francisco, CA, USA). Animals in the vehicle group were injected with saline (1 ml/kg). To assess the protein expression of TLR4 in the hippocampus, hippocampal tissue was collected for western blot analysis. During the experiments, solutions and drugs were stored at 4°C, while the antibodies were stored at −20°C.

### Procedures and Behavioral Apparatus

The general procedure of this experiment was conducted as previously described elsewhere, with slight modifications (Malvaez et al., 2010). In brief, the complete experimental procedures mainly comprise the following six phases: preconditioning phase (habituation), pretest, conditioning (CPP training), post-conditioning test (CPP test), extinction, and reinstatement. The behavioral procedures and tests were performed in a shuttle plexiglass box consisting of two equal-size compartments (24 cm length × 14 cm width × 30 cm height) with distinct patterns on floors and walls, which are connected by a smaller neutral compartment (7.0 cm length × 7.0 cm width × 30 cm height) with a removable guillotine door. One larger black compartment contained four black walls and a smooth black floor, while the other larger white compartment had four white walls and a rough white floor decorated with blue sandpaper. The smaller neutral compartment was a gray central corridor with two gray walls and a smooth gray floor. Briefly, all shuttle boxes presented different color and wall textures to pair distinct tactile and visual cues with cocaine or saline injections. The retractable guillotine doors were raised or closed according to the practical requirement of different experiments. For example, these doors were raised to allow animals to freely access any chamber when they underwent a CPP test, habituation, and non-confined extinction training. By contrast, the retractable guillotine doors were closed to prevent the animals from crossing the chambers when they underwent the training of CPP and confined extinction. An infrared camera was suspended approximately 1 m above the shuttle plexiglass box, and was used to keep track of the real-time positions of the animals throughout the three compartments. The total time and distance traveled in each compartment were recorded using a computerized video tracking system (video behavior analysis software Smart 3.0; Panlab, Spain). According to previous literature, the white compartment (non-preferred compartment) was defined as the cocaine-paired compartment and the black one (preferred compartment) was named as saline-paired compartment (Malvaez et al., 2010). The CPP score was calculated as the time spent in the cocaine-paired chamber during the test of 20 min. None of the animals died during the experiment.

### Pre-conditioning Stage (Day 1–3)

On days 1 and 2, the guillotine doors were raised and all mice were positioned in the middle of the smaller neutral chambers and were provided free access to the entire chambers for 45 min before the commencement of the pretest. The major purpose of habituation was to prepare animals for the subsequent pretest. On day 3, the pretest was initiated and baseline preferences were assessed by placing the animals in the middle of the intersection of the place preference apparatus with the guillotine doors open, allowing free access to all compartments for 20 min. The time spent in each chamber was recorded, and the time spent in the white chamber (non-preferred chamber) was chosen to determine their baseline preference. There was no drug treatment during the whole preconditioning stage. The pretest of habituation was also used to discard biased mice that spent more than 960 s and less than 240 s in any chamber (Northcutt et al., 2015), and the rest were employed to complete the following experiments. These biased animals discarded in the pretest were required to the same behavioral procedures as these unbiased ones but free of all CPP tests, and treated with saline alone during the following experiments. In order to minimize the number of mice in our study, they were finally included in the saline group used to compare to the vehicle and Ex4 groups in subsequent western blots.

### Conditioning Stage (Day 4–11)

Acquisition training of cocaine-associated CPP was implemented from day 4 to day 11. Cocaine conditioning was composed of four cocaine sessions and four saline sessions. On days 4, 6, 8, and 10, each mouse was given a systemic injection of cocaine (20 mg/kg, i.p.) and then immediately confined to one of the designed cocaine-paired chambers (non-preferred chamber) for 45 min. On days 5, 7, 9, and 11, they received an intraperitoneal injection of equivalent saline to that of cocaine and were confined to the opposite chamber (preferred chambers) for 45 min. In this phase, the guillotine doors were closed and then these mice were immediately confined in the designed cocaine- or saline-paired chambers for 45 min (Malvaez et al., 2010). Cocaine-mediated CPP training was conducted once a day. After the completion of daily CPP training, all mice were returned to their home cage.

### Post-conditioning Test (Day 12)

Following 8 days of the cocaine-induced CPP training, the guillotine doors were raised and the room preference test was initiated. On the post-test day, these mice were positioned in the center of the smaller neutral chamber and allowed to freely explore the whole conditioning apparatus for 20 min. No drug was administered on the test day. The CPP score was calculated as the time spent in the cocaine-paired chamber during the test of 20 min. The cocaine-mediated CPP was considered to be successfully achieved when the CPP score was significantly higher in the post-test than in the pretest. To test the extinction of drug-associative preference behavior, mice with CPP scores more than 960 s or less than 240 s in the cocaine-paired chamber were considered to have no preference and were excluded from the next study. After the completion of the CPP test, these mice were randomly split into two groups to experience daily extinction training.

### CPP Extinction (Days 11–19)

The day after the completion of the training and test of cocaine-induced CPP, extinction training was initiated in response to the requirements of different experiments.

**Figure 1:**
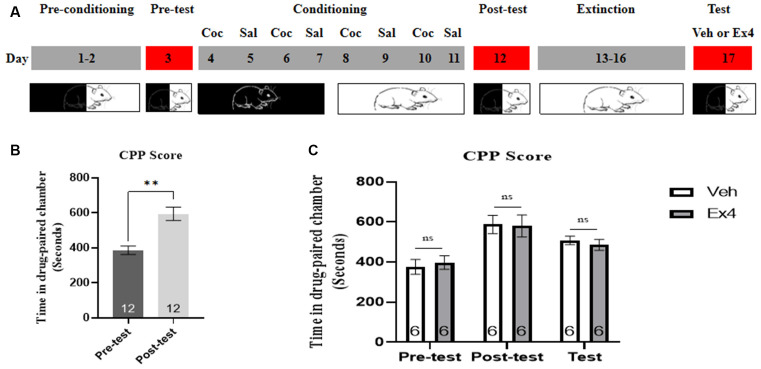
Ex4’s single treatment fails to enhance the confined extinction of cocaine-induced CPP. **(A)** The experimental schedule for confined extinction procedures and saline (Sal), cocaine (Coc), as well as extendin4 (Ex4) treatment protocols. **(B)** CPP scores of pre-test and post-test indicate that most animals showed a significant place preference following cocaine-paired CPP training. *N* = 12 per group, **represents *p* < 0.01 vs. pretest, paired *t*-test. **(C)** CPP scores of vehicle or Ex4 suggest that after four confined extinction sessions single treatment with Ex4 on the test day failed to induce a significant reduction in cocaine-induced CPP scores compared to vehicle. *N* = 6 per group, ns represents no significance, Student’s *t*-test. Data are expressed as mean ± SEM. CPP, condition place preference.

**Experiment 1**: For the next four consecutive confined extinction sessions, the guillotine doors were closed and all animals were confined in the designated cocaine-paired chamber for 45 min and underwent daily extinction training for 4 days (day 13–16). There was no cocaine treatment during the four consecutive extinction sessions. Subsequently, they were randomly divided into two independent groups. On the extinction test day, half of them received an acute single injection Ex4 (1.0 μg/kg, i.p.) dissolved in saline and half received vehicle (equivalent saline to that of Ex4, 1.0 ml/kg, i.p.). After that, the guillotine doors were raised and all of them were allowed to freely explore the three chambers for 20 min. The effect of a single Ex4 on the extinction response to cocaine-induced CPP was assessed after each 4-day period of extinction training during an extinction test on day 17 (Figure 1A). After completion of this test, mice were returned to their home cages.

**Figure 2:**
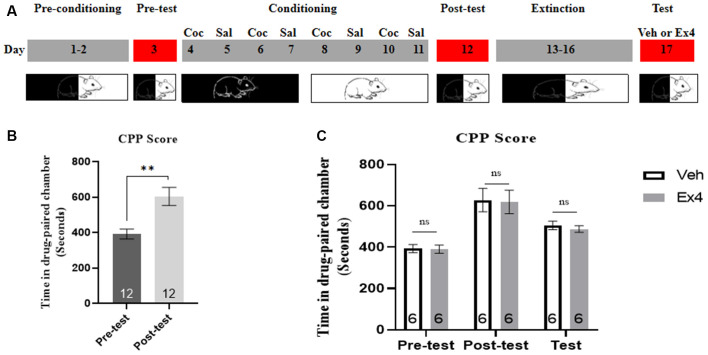
Ex4’s single treatment fails to potentiate the non-confined extinction of cocaine-induced CPP. **(A)** The experimental schedule for non-confined extinction procedures and saline (sal), cocaine (Coc), as well as exendin4 (Ex4) treatment protocols. **(B)** CPP scores of pre-test and post-test indicate that most animals showed a significant place preference following cocaine-paired CPP training. *N* = 12 per group, **represents *p* < 0.01 vs. pretest, paired *t*-test. **(C)** CPP scores of vehicle or Ex4 suggest that after four non-confined extinction sessions single treatment with Ex4 on the test day failed to induce a significant reduction in cocaine-induced CPP scores compared to vehicle. *N* = 6 per group, ns represents no significance, Student’s *t*-test. Data are expressed as mean ± SEM.

**Experiment 2**: For the next four consecutive non-confined extinction sessions, we repeated Experiment 1 using similar behavioral procedures and treatment timeline, with the exception that the guillotine doors were raised and animals had free access to any chamber for 45 min during each extinction session. Animals experienced the same procedures on the test day as Experiment 1 (Figure 2A).

**Figure 3:**
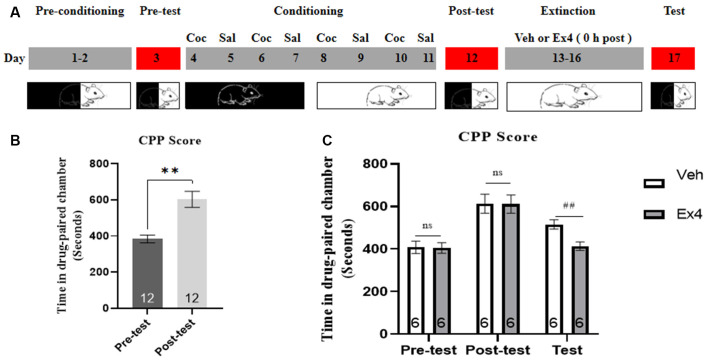
Ex4’s treatment 0 h after extinction session drives the confined of cocaine-induced CPP. **(A)** The experimental schedule for confined extinction procedures and saline (Sal), cocaine (Coc), as well as extendin4 (Ex4) treatment protocols. **(B)** CPP scores of pre-test and post-test indicate that most animals showed a significant place preference following cocaine-paired CPP training. *N* = 12 per group, **represents *p* < 0.01 vs. pretest, paired *t*-test. **(C)** CPP scores of vehicle or Ex4 suggest that after four confined extinction sessions treatment with Ex4 0 h after each extinction session induced a significant reduction in cocaine-induced CPP scores compared to vehicle. *N* = 6 per group, ^##^represents *p* < 0.01, Student’s *t*-test. Data are expressed as mean ± SEM.

**Experiment 3**: After the completion of the CPP test, the animals were split into two groups and then underwent daily extinction training for 4 days (day 13–16). For the next 4 consecutive days of confined extinction sessions, the guillotine doors were closed and all animals were confined in the designed cocaine-paired chamber for 45 min and underwent daily extinction training for 4 days (day 13–16). During this time, half of the animals were given a daily injection of Ex4 (1.0 μg/kg, i.p.) dissolved in saline and half were given a daily injection of vehicle (equivalent saline to that of Ex4, 1.0 ml/kg, i.p.) immediately after exposure to the previously cocaine-paired chamber. There was no cocaine treatment during the following four consecutive extinction sessions. On the extinction test day, the guillotine doors were raised and these animals were subject to a room preference test 24 h after four days of confined extinction sessions. The effect of repeated Ex4 immediately after each extinction training on the extinction response to cocaine-induced CPP was assessed after each 4-day period of extinction training during an extinction test on day 17 (Figure 3A). There was no treatment on test day. After completion of this test, mice were returned to their home cages.

**Figure 4:**
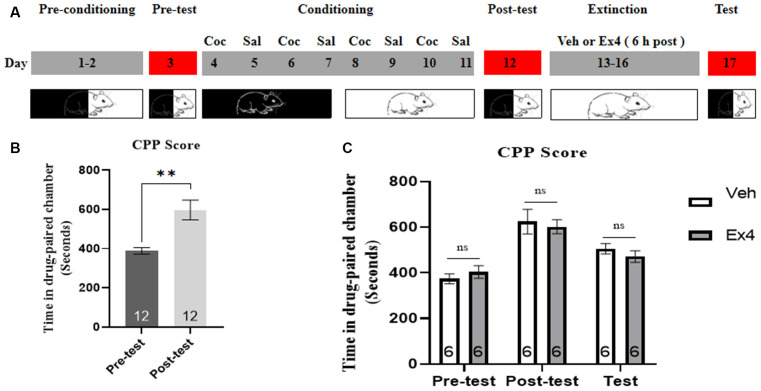
Ex4’s treatment 6 h after each extinction fails to facilitate the confined extinction of cocaine-induced CPP. **(A)** The experimental schedule for confined extinction procedures and saline (Sal), cocaine (Coc), as well as extendin4 (Ex4) treatment protocols. **(B)** CPP scores of pre-test and post-test indicate that most animals showed a significant place preference following cocaine-paired CPP training. *N* = 12 per group, **represents *p* < 0.01 vs. pretest, paired *t*-test. **(C)** CPP scores of vehicle or Ex4 suggest that after four confined extinction sessions treatment with Ex4 6 h after each extinction session failed to induce a significant reduction in cocaine-induce CPP scores compared to vehicle. *N* = 6 per group, ns represents no significance, Student’s *t*-test. Data are expressed as mean ± SEM.

**Experiment 4**: Mice were subject to similar procedures and timeline to that of Experiment 3, except that they received an injection of either Ex4 or saline 6 h after daily extinction training. The timeline for Experiment 4 was shown in Figure 4A. The mice underwent the same procedures of behavioral test day as Experiment 3.

**Figure 5:**
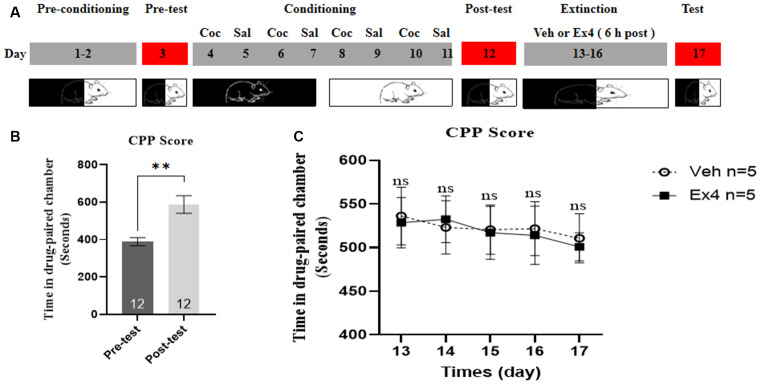
Ex4’s treatment 6 h after each extinction fails to promote non-confined extinction of cocaine-induced CPP. **(A)** The experimental schedule for non-confined extinction procedures and saline (Sal), cocaine (Coc), as well as extendin4 (Ex4) treatment protocols. **(B)** CPP scores of pre-test and post-test indicate that most animals showed a significant place preference following cocaine-associated CPP training. *N* = 12 per group, **represents *p* < 0.01 paired *t*-test. **(C)** CPP scores of vehicle or Ex4 suggest that during four non-confined extinction sessions treatment with Ex4 6 h after each extinction session failed to induce a significant reduction in cocaine-induce CPP score compared to vehicle. *N* = 6 per group, ns represents no significance, two-way rmANOVA. Data are expressed as mean ± SEM.

**Experiment 5**: For the next four consecutive non-confined extinction sessions, the guillotine doors were raised, allowing animals free access to any chambers. For the following 4 days (day 13–16), mice were subject to a room preference test every day (20 min, non-confined extinction), followed by either a daily injection of Ex4 (1.0 μg/kg, i.p.) or a daily injection of equivalent saline to that of Ex4 (1.0 ml/kg, i.p.), 6 h after exposure to the conditioned chambers for 20 min. No cocaine was administrated during the whole extinction sessions. After finishing the entire extinction, the guillotine doors were raised and animals experienced a room preference test of 20 min. The effect of repeated Ex4 6 h after each extinction training on the extinction response to cocaine-induced CPP was assessed after each 4-day period of extinction training during an extinction test on day 17 (Figure 5A). After this test, mice were put back into their cages.

**Figure 6:**
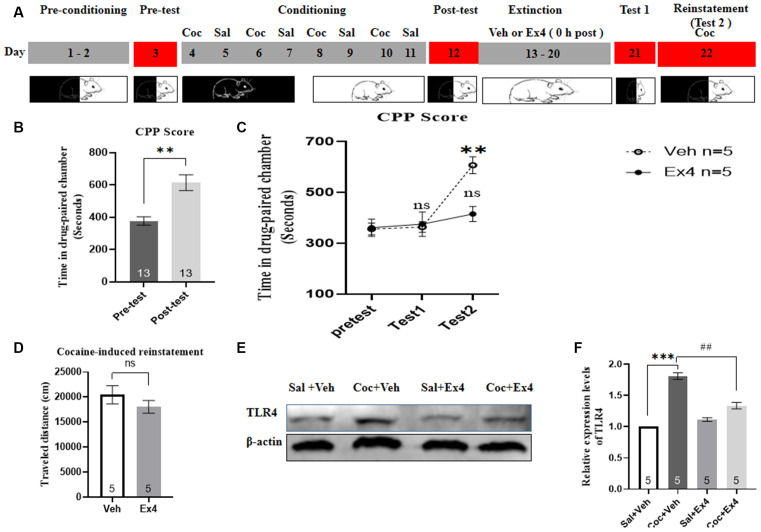
Ex4’s treatment 0 h after each extinction attenuates reinstatement of cocaine-induced CPP. **(A)** The experimental schedule for confined extinction procedures and saline (Sal), cocaine (Coc), as well as exendin4 (Ex4) treatment protocals. **(B)** CPP scores of pre-test and post-test indicate that most animals showed a significant place preference following cocaine-associated CPP training. *N* = 13 per group, **represents *p* < 0.01, paired *t*-test. **(C)** CPP scores after systemic administration of vehicle and Ex4 show that Ex4attenuated reinstatement. *N* = 5 per group, **represents *p* < 0.01 vs. pretest, ns represents no significance, two-way ANOVA. **(D)** Distance traveled of vehicle and Ex4 on the cocaine-primed reinstatement test day. ns represents no significance vs. vehicle group, Student’s *t*-test. **(E)** Representative image of TLR4 expression in the saline, vehicle and Ex4 groups detected by Western blot analysis. **(F)** Semiquantitative analysis of the relative levels of TLR4 in hippocampus by densitometric analysis. *N* = 5 per group, ***represents *p* < 0.001 vs. Sal group; ^##^represents *p* < 0.01 vs. vehicle group, one-way ANOVA followed by Bonferroni’s *post hoc* test. Date are expressed as mean ± SEM.

**Experiment 6**: After the completion of the CPP test, the animals were divided into two groups and then underwent daily extinction training for 8 days (Figure 6A). For the next eight consecutive confined extinction sessions, the guillotine doors were closed, stopping animals from crossing the conditioned chambers. During the whole cocaine-free extinction sessions, mice received a daily injection of Ex4 (1.0 μg/kg, i.p.) or daily injections of vehicle (equivalent saline to that of Ex4, 1.0 ml/kg, i.p.) immediately after exposure to the previously designated cocaine-paired chamber for 45 min once a day. No cocaine was injected during this period. Afterward, the effect of chronic Ex4 on the extinction response to cocaine-induced CPP was evaluated in a drug-free state after 8-days confined extinction training in an extinction test on day 21, and then were returned to their plastic cage. In this phase, extinction criterion was considered to be achieved when there was no significant difference in CPP scores in comparison with the pretest. After the completion of the final extinction test, animals were given a cocaine-primed reinstatement test. These mice that achieved extinction criterion were reinstated with a lower dose of cocaine (10 mg/kg, i.p.) before the commencement of the reinstatement test. On the reinstatement test day, the guillotine doors were raised, allowing animals free access to each chamber. After completing the reinstatement test, mice were euthanized by cervical dislocation, and their brain tissues were immediately collected for TLR4 Western blotting.

**Figure 7:**
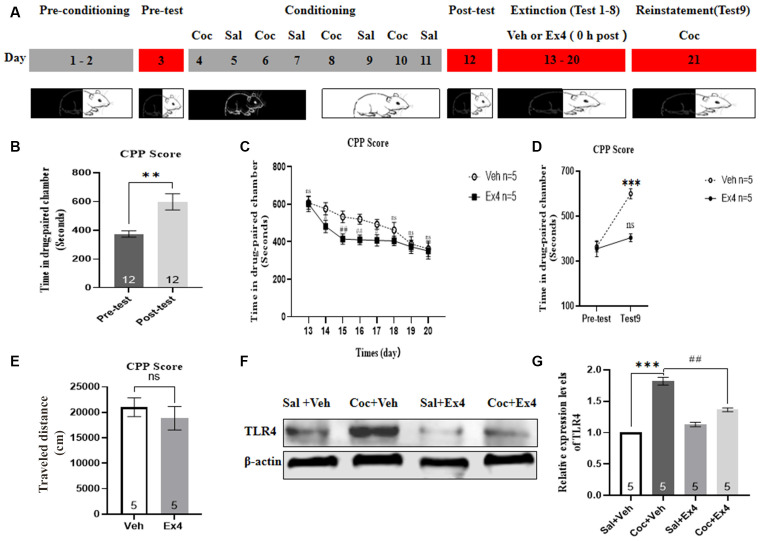
Ex4’s repeated treatment 0 h after each extinction enhances the non-confined extinction of cocaine-induced CPP and blocks cocaine-primed reinstatement of cocaine-induced CPP. **(A)** Timeline of behavioral testing and pharmacologic treatment protocals. **(B)** CPP scores of pre-test and post-test, indicated that most animals displayed a place preference for the cocaine-paired compartment following cocaine-induced CPP training. **represents *p* < 0.01 vs. pretestby Student’s *t*-test. **(C)** CPP scores of systemic administration of vehicle or Ex4 0 h after each extinction show that Ex4 promoted the early extinctioin in mice. *N* = 5 per group, ^#^ and ^##^represents *p* < 0.05 and *p* < 0.01 vs. vehicle group, respectively, ns represents no significance, two-way ANOVA. **(D)** CPP scores after treatment with vehicle or Ex4 indicate that Ex4 inhibited the reinstatement of cocaine-induced CPP. ***represents *p* < 0.001 vs. pretest, ns represents no significance, two-way AVOVA. **(E)** Distance traveled of vehicle and Ex4 on the cocaine-primed reinstatement test day. *N* = 5, ns represents no significance vs. vehicle group, Student’s t-test. **(F)** Representative image of TLR4 expression detected by Western blot analysis. **(G)** Semiquatitative analysis of the relative levels of TLR4 by densitometric analysis. ***represents *p* < 0.001 vs. Sal group, ^##^represents *p* < 0.01 vs. vehicle group by one-way ANOVA. Data are mean ± SEM. Coc, cocaine; Sal, saline; Ex4, exendin-4; Veh, vehicle.

**Experiment 7**: For the next eight consecutive non-confined extinction sessions, the guillotine doors were raised, allowing animals free access to any chambers (Figure 7A). For the following 8 days (day 13–20), mice were subject to a room preference test every day (20 min, non-confined extinction), followed by either a daily injection of Ex4 (1.0 μg/kg, i.p.) or a daily injection of saline (1.0 ml/kg, i.p.) immediately after exposure to the conditioned chambers for 20 min. No cocaine was administrated during the whole extinction sessions. Afterward, the effect of chronic Ex4 on the extinction response to cocaine-induced CPP was evaluated in a drug-free state after eight non-confined extinction training in an extinction test on day 17, and then they were returned to their vivariums. In this phase, CPP was considered to be extinguished when there was no significant difference in CPP scores compared to the pretest. Following the completion of all extinction experiments, these mice that achieved extinction criterion were primed with a lower dose of cocaine (10 mg/kg, i.p.) prior to the initiation of the reinstatement test. On the reinstatement test day, the guillotine doors were raised, and animals were allowed to freely explore each chamber again. After completing the reinstatement test, mice were euthanized by cervical dislocation, and their brains were immediately removed for TLR4 Western blotting.

### Locomotor Activity

Locomotor activity as the total distance traveled in each chamber on the reinstatement test day was recorded while the animals were placed in the CPP apparatus.

### Western Blot Analysis

Western blot analysis was used to detect the expression levels of TLR4 in the hippocampus. Mice from each group were sacrificed by decapitation 1 h after the cocaine-primed reinstatement procedure, and the whole hippocampal tissues of five animals from each group were harvested and homogenized in radio immunoprecipitation assay (RIPA) lysis buffer and then centrifuged at 12,000 *g* for 5 min at 4°C. Rhe supernatants of whole hippocampal tissues were collected and extracted using the BCA Protein Extraction Kit (KGP2100, KeyGEN Biotechnology Co., Ltd, Jiangsu, China). Protein concentration was determined using the BCA Protein Assay Kit (KGP902, KeyGEN Biotechnology Co., Ltd, Jiangsu, China). The hippocampal tissue of the mice was placed on the ice during all experiments. Equal amounts of protein (50 μg per lane) were resolved on a 10% or 12% sodium dodecyl sulfate–polyacrylamide gel (SDS-PAGE), and then transferred onto 0.22-μm polyvinylidene fluoride (PVDF) membranes (Millipore, USA) blocked with 5% non-fat milk. The PVDF membranes were incubated with the following primary antibodies overnight at 4°C: rabbit anti-TLR4 (1:1,000) and β-actin (1:2,000). Membranes were washed with TBS containing 1% Tween 20 three times, and subsequently incubated with the corresponding secondary antibody (1:5,000) for 1 h at room temperature. Detection was performed using an Odyssey Infrared Imaging System CLX-0796 (LI-COR, Lincoln, NE, USA). The protein bands were quantified *via* densitometrical analysis using Image-Pro Plus 6.0 software (Media Cybernetics, Inc., Rockville, MD, USA). The ratio of the gray value of the target protein band to that of the saline group was used to determine the relative expression levels of the target protein. All experiments were performed in triplicate. Normalized methods were applied for western blot quantification to reduce the influence of variations arising from experimental error. In detail, the stained blot was imaged, a line was drawn around the target protein TLR4 and β-actin in each lane, and then the gray values of target protein band density were analyzed using Image J software. The relative value of each of the target protein expression levels was presented as the gray values of each target protein divided by the corresponding β-actin. In this way, the relative gray values of the saline, vehicle, and Ex4 groups were obtained. Subsequently, the relative gray values of different groups were divided by the saline group. At last, the normalizing western blot data of the protein in each group was procured.

### Statistical Analysis for Experiment 1–4

Statistical analyses were performed using GraphPad 8.4 software. Data are presented as mean ± standard errors of the means (SEM). The data from cocaine-induced CPP between pretest and post-test were analyzed using paired *t*-test. A Student’s *t*-test was used to compare two independent groups. One-way analysis of variance (ANOVA) was performed to compare more than two groups. Bonferroni *post hoc* analysis was subsequently performed after one-way ANOVA or two-way repeated-measures ANOVA within different factors. The threshold for statistical significance was *p* < 0.05 (GraphPad, v.8.4, California, USA).

## Results

### Impact of Single Administration of Ex4 on Confined Extinction of Cocaine-Induced Condition Place Preference (CPP)

We first tested the behavioral actions of a single injection of Ex4 on the confined extinction of cocaine-induced CPP. On the pretest day, a total of three mice were discarded because they had a particularly strong preference for any of the chambers (>960 s in the white or black chamber). Then the rest were required to undergo 8 days of CPP training. No mice were excluded due to training failure of the cocaine-related CPP paradigm. As shown in Figure 1B, all animals spent much more time in the cocaine-paired chamber after 8 days of CPP training when compared to the pretest (*t_11_* = 4.379, *p* = 0.0011, paired *t*-test), indicating that the initial memory or association between cocaine and chamber has been successfully established. Subsequently, animals were randomly split into two groups and Ex4 (1 μg/kg, i.p.) or vehicle (saline, 1 ml/kg, i.p.) were administered on the extinction test day. In the subsequent drug-free CPP test after four consecutive confined extinction sessions, however, Student’s *t*-test showed that Ex4 failed to induce a significant reduction in CPP score when compared to the vehicle group (*t_10_* = 1.146, *p* = 0.2783, Student’s *t*-test; Figure 1C). Consequently, these results suggested that an acute single administration of Ex4 on the extinction test day can not enhance the confined extinction of cocaine-induced CPP.

### Impact of Single Administration of Ex4 on Non-confined Extinction of Cocaine-Induced Condition Place Preference (CPP)

We then gained insight into the impact of Ex4 on non-confined extinction. On the pretest day, there was no mouse was discarded because of a particularly strong preference for any of the chambers. Then all animals were required to experience 8 days of CPP training. During this trial, three mice were excluded due to the CPP training failure. As shown in Figure 2B, most animals developed a significant preference for the cocaine-paired chamber when compared to the pretest (*t_11_* = 3.790, *p* = 0.0030, paired *t*-test), implying that the initial cocaine-environment memory was well set. After then, animals were randomly divided into two groups and Ex4 (1.0 μg/kg, i.p.) or vehicle (saline, 1 ml/kg, i.p.) were given on the extinction test day. During the follow-up CPP tests, the time spent in the cocaine-paired chamber was not shortened by Ex4 treatment when compared to the vehicle group mice (*t_10_* = 1.320, *p* = 0.2195, Student’s *t*-test; Figure 2C). Taken together, these data suggested that an acute single administration of Ex4 on the extinction test day can not potentiate the non-confined extinction of cocaine-induced CPP.

### Impact of Repeated Administration of Ex4 Immediate After Daily Extinction Session on Confined Extinction of Cocaine-Induced CPP

We further investigated the influence of daily injection of Ex4 0 h after each extinction training on the confined extinction of cocaine-induced CPP. In the pretest, no mouse was discarded because of a particularly strong preference. Then 8 days of CPP training was initiated. During this period, only one mouse was excluded because of the failure of CPP training. 12 animals showed a significant room preference for the cocaine-paired chamber following conditioning when compared to the pretest situation (*t*_11_ = 4.234, *p* = 0.0014, paired *t*-test; Figure 3B). Subsequently, all animals were randomly separated into two groups and Ex4 (1.0 μg/kg, i.p.) or vehicle (saline, 1 ml/kg, i.p.) were given 0 h after re-exposure to the previously drug-paired compartment on the four consecutive days of confined extinction sessions. Afterward, another place preference test was performed. A Student’s *t*-test showed that repeated administration of Ex4 can reduce the CPP score compared to the vehicle group (*t*_10_ = 3.624, *p* = 0.0047; Figure 3C). Thus, these results indicated that treatment with Ex4 immediately after extinction training drives the confined extinction of cocaine-induced CPP.

### Impact of Repeated Administration of Ex4 6 h After Daily Extinction Session on Confined Extinction of Cocaine-Induced CPP

We measured the effect of daily injection of Ex4 6 h after re-exposure to the previously drug-paired compartment on the confined extinction of cocaine-induced CPP. In the pretest, no mouse was discarded because of a particularly strong preference. Then these animals were subjected to 8 days of CPP training. During this period, two mice were discarded because of CPP training failure. As shown in Figure 4B, the time spent of most animals in the cocaine-paired chamber was significantly increased following cocaine-conditioning when compared to the pretest situation (*t*_11_ = 3.735, *p* = 0.0033, paired *t*-test). Subsequently, all animals were randomly separated into two groups and exposed to four consecutive confined extinction of CPP. However, Student’s *t*-test showed that the administration of Ex4 6 h after each extinction training failed to decrease the CPP score (*t*_10_ = 1.215, *p* > 0.05; Figure 4C). Briefly, these results revealed that treatment with Ex4 6 h after extinction training could not enhance the confined extinction of cocaine-induced CPP.

### Impact of Repeated Administration of Ex4 6 h After Daily Extinction Session on Non-confined Extinction of Cocaine-Induced CPP

We further assessed the impact of Ex4 on the non-confined extinction. During the stage of pretest and post test, no mice werediscarded. These animals were required to receive 8 days of CPP training. As shown in Figure 5B, the time spent in the cocaine-paired chamber was significantly longer in the post-conditioning stage than in the pretest stage (*t*_11_ = 4.177, *p* = 0.0015, paired *t*-test). After that, all animals were randomly separated into two groups and experienced the following four consecutive non-confined extinction of CPP. Two-way rmANOVA revealed that there was no statistical difference in extinction CPP scores between vehicle-treated mice and Ex4-treated mice across days (treatment: *F*_(1,20)_ = 0.0344, *p* = 0.8546; test session: *F*_(4,20)_ = 0.3579, *p* = 0.8355; treatment × test session interactions *F*_(4,20)_ = 0.02728, *p* = 0.9984; Figure 5C), indicating that repeated administration of Ex4 6 h after extinction training had no significant effect on early non-confined extinction when compared to the vehicle group. Altogether, these results illustrated that Ex4 treatment 6 h after each extinction training could not promote non-confined extinction of cocaine-induced CPP.

### Impact of Repeated Administration of Ex4 Immediately After Daily Extinction Session on Confined Extinction and Reinstatement of the Cocaine-Induced CPP

We next evaluated the role of Ex4 in the confined extinction and reinstatement of the cocaine-induced CPP. In this trial, animals first needed to complete the habituation and pretest, and then they underwent 8 days of CPP training and test. Four out of 14 animals were discarded because of particularly strong preference or CPP training failure. As shown in Figure 6B, a paired *t*-test revealed that mice presented preference for the cocaine-paired compartment following CPP training when compared to the pretest (*t*_11_ = 3.764, *p* = 0.0027, paired *t*-test). Subsequently, these animals that completed the CPP test were randomly divided into two independent groups to receive systemic injections of Ex4 (1.0 μg/kg, i.p.) or vehicle (saline, 1.0 ml/kg, i.p.) 0 h after each confined extinction session, and then they were given a cocaine-primed reinstatement test evoked by an injection of 10 mg/kg of cocaine to further assess the sustained effects of extinction. A two-way rmANOVA revealed a statistical difference in CPP scores between vehicle-treated mice and Ex4-treated mice (treatment: *F*_(1,8)_ = 10.46, *p* = 0.0120; test session: *F*_(2,8)_ = 10.86, *p* = 0.0010; treatment × test session interactions *F*_(2,64)_ = 5.011, *p* = 0.0204; Figure 6C). Furthermore, two-way rmANOVA followed by Bonferroni’s multiple comparisons *post hoc* analysis suggested that there is no significant difference in CPP scores compared to the baseline preference (*p* > 0.05 for Ex4 and vehicle groups, two-way rmANOVA; Figure 6C), indicating that they achieved extinction criteria. By contrast, two-way rmANOVA followed by Bonferroni’s multiple comparisons *post hoc* analysis showed that vehicle-treated mice produce a significant increase in CPP scores compared with the baseline preference (*p* = 0.0014; Figure 6C), implying that cocaine (10 mg/kg) successfully induced a reinstatement of CPP. Moreover, Bonferroni’s multiple comparisons *post hoc* analysis showed that Ex4-treated mice failed to induce a significant reduction in CPP scores compared to the baseline preference (*p* > 0.05; Figure 6C), indicating that Ex4 reduced the reinstatement of CPP. Additionally, the Student’s *t*-test showed that there was no inhibitory effect of Ex4 treatment on locomotor activity during the reinstatement state (*t*_10_ = 1.085, *p* = 0.0674; Figure 6D). To conclude, these results indicated that daily administration of Ex4 immediately after each extinction session facilitates the confined extinction and attenuates reinstatement of cocaine-induced CPP.

To evaluate the ability of Ex4 to down-regulate the expression of TLR4 in the hippocampus, we isolated this area of the brain and analyzed TLR4 levels using Western blotting. The hippocampus was chosen for the analysis because it is the center of learning and memory in mammals (Bohbot and Corkin, 2007). As depicted in Figures 6E,F the saline, vehicle, and Ex4 groups displayed a significant difference in the relative expression levels of TLR4 by Western blotting (*F*_(3,19)_ = 82.52, *p* = 0.0006, one-way ANOVA), and then a Bonferroni’s multiple comparison *post hoc* analysis showed that the relative protein content of TLR4 was higher in the vehicle group than in the Ex4 group (*p* = 0.0066). Together, these data revealed that repeated post-extinction administration of Ex4 alleviates the release of TLR4 within the hippocampus.

### Impact of Repeated Administration of Ex4 0 h After Daily Extinction Session on Non-confined Extinction and Reinstatement of the Cocaine-Induced CPP

We finally examined the effect of repeated administration of Ex4 on the non-confined extinction and reinstatement of cocaine-induced CPP. In this trial, animals firstly completed the habituation and pretest, and then they were required to undergo 8 days of CPP training and test. Five out of 15 animals were ruled out due to either particularly strong natural preference or CPP acquisition failure. Consistent with previous results, animals had higher CPP scores following eight consecutive cocaine-conditioning training sessions in comparison with the pretest (*t*_11_ = 3.627, *p* = 0.0040, paired *t*-test; Figure 7B), clearly revealing that over the course of the establishment of the CPP paradigm, associations or memories were successfully established between environmental cues and the rewarding effects of cocaine, and then contributed to a place preference for the cocaine-paired environment. Subsequently, these animals were randomly divided into two groups and received systemic injections of Ex4 (1 μg/kg, i.p.) or vehicle (saline, 1.0 ml/kg, i.p.) immediately after eight non-confined extinction sessions of CPP. During drug-free room preference tests, which served as extinction sessions, the two-way rmANOA revealed a significant difference in CPP scores among saline, vehicle and Ex4-treated groups (treatment: *F*_(1,8)_ = 57.13, *p* < 0.001; test session: *F*_(7,8)_ = 65.48, *p* < 0.001; and treatment × test session interactions: *F*_(7,64)_ = 4.345, *p* < 0.001; Figure 7C). Additionally, the Bonferroni’s multiple *post hoc* analysis showed that in test 2, 3, 4, and test 5 (herein referred to as the first 4 days of extinction training), mice treated with Ex4 (1.0 μg/kg) spent less time in the cocaine-paired compartment when compared to vehicle-treated mice (*p*_(test 2)_ = 0.0134; *p*_(test 3)_ = 0.0041; *p*_(test 4)_ = 0.0012; *p*_(test 5)_ = 0.00137; Figure 7C). These results indicated that Ex4 immediately after daily extinction training (during the first four extinction sessions) acts as a post-extinction pharmacological strategy to promote the early extinction. However, there was no statistical significance in CPP scores among Test 6, 7, and 8 when compared to the vehicle group (*p* > 0.05, two-way rmANOA; Figure 7C), implying that following eight extinction sessions the cocaine-induced CPP was completely extinguished in all vehicle and Ex4 treatment groups during this period. Collectedly, these results showed that the combination of Ex4 with extinction training enhances the extinction of cocaine-induced CPP. To explore the persisting effects of Ex4 on extinction, animals were subjected to a reinstatement test evoked by half-dose of cocaine (10 mg/kg) after the last extinction session. A two-way rmANOVA suggested that there was a significant difference in CPP scores between vehicle and Ex4 groups (treatment: *F*_(1,8)_ = 24.13, *p* = 0.0012; test session: *F*_(1,8)_ = 23.43, *p* = 0.0013; and treatment × test session interactions: *F*_(1,64)_ = 10.08, *p* = 0.0131; Figure 7D). Furthermore, Bonferroni’s multiple comparisons *post hoc* analysis showed that mice in the vehicle group produced a significant increase in CPP scores compared to the baseline preference (*p* = 0.0009, two-way rmANOVA; Figure 7D), cocaine (10 mg/kg) induced a significant reinstatement of CPP. By contrast, Bonferroni’s multiple comparisons *post hoc* analysis showed that mice in Ex4 group failed to produce a significant reduction in CPP scores compared to the baseline preference (*p* = 0.5452, two-way rmANOVA; Figure 7D) demonstrating that Ex4 successfully inhibited the cocaine-primed reinstatement. However, Ex4 treatment (1.0 μg/kg) had no effect on locomotion (*t_8_* = 2.039, *p* = 0.078, Student’s *t*-test; Figure 7E). Altogether, these results of the present study confirmed that repeated administration of Ex4 as a post-extinction intervention blocks cocaine-primed reinstatement of cocaine-induced CPP.

After completing all behavioral procedures, animals were sacrificed and the hippocampus tissues were collected to detect the expression levels of TLR4 using Western blot. As shown in Figures 7F,G, there was a statistical difference in relative protein content of TLR4 among these groups (*F*_(3,19)_ = 107.7, *p* < 0.001, one-way ANOVA), and the expression levels of TLR4 within the hippocampus were significantly decreased in the Ex4-treated groups than in the vehicle group (*p* = 0.0081, Bonferroni’s multiple comparison test). Overall, these data revealed that repeated post-extinction administration of Ex4 minimizes the expression levels of TLR4.

## Discussion

In the present study, we demonstrated the vital role of glucagon-like peptide-1 receptor agonist exendin-4 (Ex4) and toll-like receptor 4 (TLR4) in the extinction and reinstatement of cocaine-induced conditioned place preference (CPP). Specifically, systemic injection of Ex4 immediately after each extinction session could not only precipitate the extinction of cocaine-induced conditioned place preference (CPP) but also attenuate the reinstatement evoked by a half-dose of cocaine (10.0 mg/kg). Moreover, we also showed that the mechanism by which GLP-1R agonist exerted inhibitory effects on the reinstatement of cocaine-induced behavior might be related to the critical time window of extinction memory consolidation. Consequently, these results also provide additional support for the clinical availability of Ex4 or other GLP-1R agonists in the treatment of cocaine abuse.

### GLP-1R Agonists and Cocaine Addiction

Recent literature indicated that GLP-1R agonist Ex4 appears highly promising in attenuating the rewarding and reinforcing effects of cocaine addiction (Hernandez and Schmidt, 2019; Zhu et al., 2021). For instance, systemic administration of Ex4 is believed to play a substantial role in cocaine dependence (Hernandez et al., 2019), such as attenuating cocaine-induced CPP, self-administration, and the cocaine-triggered relapse of drug-seeking in an animal model of cocaine use disorder (Engel and Jerlhag, 2014; Hayes and Schmidt, 2016; Hernandez et al., 2018; Hernandez and Schmidt, 2019). Our results extend this research and identified a vital role of GLP-1R agonist Ex4 in extinction and reinstatement in a preclinical model of chronic cocaine exposure.

Our work also found that the overwhelming majority of animals that repeatedly received administration of cocaine during CPP training on alternate days developed a place preference for the cocaine-paired environment. This is consistent with previous studies suggesting that 20 mg/kg of cocaine was sufficient to induce CPP paradigm (Meye et al., 2016; Ladrón de Guevara-Miranda et al., 2019) which has been commonly considered as a popular animal model for studying the rewarding effects of addictive drugs (Bardo and Bevins, 2000; Prus et al., 2009). This implies that the rewarding properties of cocaine (unconditioned stimulus) were well paired with the white chamber (conditioned stimuli) during the conditioning phase, and then the original cocaine-environment memories or associations were successfully established. Once these memories are established, they would enter a short period of destabilization when they are liable to cocaine craving and relapse following a prolonged period of abstinence (Torregrossa and Taylor, 2013). However, if there is an approved intervention in the destabilization period, the original drug-associated memory would not enter long-term storage throughout restabilization or reconsolidation (Sorg, 2012), thereby reducing the susceptibility to relapse. Indeed, manipulating memory reconsolidation by extinction training has also been proposed as a therapeutic alternative to decrease the risk of relapse of cocaine addiction (Rich and Torregrossa, 2018). For instance, many pharmacological agents including garcinol (Dunbar and Taylor, 2017; Monsey et al., 2017) and cannabidiol (Carvalho and Takahashi, 2017) repeatedly exert critical roles in the memory of cocaine abuse. Similarly, extensive studies have also proven that post-extinction pharmacological interventions effectively enhance the extinction of drug-associated behavior (Zhou and Kalivas, 2008; Chesworth and Corbit, 2015). Therefore, weakening the strength of these cocaine-context memories may be an available intervention for enhancing lasting abstinence (Deębiec et al., 2004; Duvarci and Nader, 2004; Kindt et al., 2009). Interestingly, GLP-1R agonists have also been reported to improve learning and memory (Gault and Hölscher, 2018). In light of these findings above, we hypothesized that Ex4 administration immediately after each extinction training during extinction consolidation sessions (herein referred to as extinction) produce new formation of learning and memories which would effectively counteract the original cocaine-associated memories, and thereby facilitating extinction and attenuate reinstatement of cocaine-primed CPP. More interestingly, our results confirmed this hypothesis, as animals that were exposed to the confined drug-paired or non-confined context in the absence of drug reward and treated with Ex4 immediately after extinction presented a lower CPP score than those in the vehicle group when they underwent the CPP test of cocaine-primed reinstatement. However, whether Ex4 treatment 6 h after daily extinction exerted the same influence on improving the maladaptive behavior of cocaine remains to be cleared. To examine this hypothesis, animals received acute single administration of Ex4 on the extinction test day or injection of Ex4 6 h after each extinction session. As a consequence, our study showed that Ex4 treatment during this period did not facilitate cocaine-induced extinction or reduce reinstatement, suggesting that extinction training is an indispensable factor for therapeutic effects of Ex4. This result was in accordance with a previous study determining that 6 h was the critical time window of memory (Kopp et al., 1966).

In addition, although the CPP score of animals from the vehicle group declined progressively during extinction, the magnitude of the effect was clearly lower than that of the Ex4 (1.0 μg/kg) treatment group in tests 2, 3, 4, and 5 of non-confined extinction, suggesting that the efficacy of exposure therapy alone is limited (Weiss et al., 2001; Torregrossa and Taylor, 2013). Furthermore, these animals in the vehicle group have proven more susceptible to reinstatement than ones in the Ex4 group, revealing that Ex4 lessened the cocaine-primed reinstatement (Hernandez et al., 2018).

Altogether, these findings showed that emerging memories induced by the combination of Ex4 treatment with extinction training suppressed the expression of initial cocaine-associated memories, and thereby weakening the ability of cocaine-associated cues to trigger craving and reinstatement after re-exposure to the cocaine-related stimuli. Thus, systemic administration of Ex4 accompanied by the daily extinction training is adequate to potentiate extinction and reduce the cocaine-induced reinstatement of CPP in an animal model of cocaine abuse. However, the associated alternations induced by cocaine and Ex4 treatment are not fully understood to date.

### Extinction and Inflammation

In general, the emerging no cocaine/cocaine cue associative memories formed during extinction training can suppress the initial cocaine-related memories to control behavior (Millan et al., 2011; McNally, 2014). Furthermore, neuroinflammation may serve as a promising target for the treatment of substance use disorder as a growing body of studies have linked neuroinflammatory markers including TLR4 to memory processes (Bader and Winklhofer, 2020). The main pathway of neuroinflammatory processes is triggered by pattern recognition receptors (PRRs), including toll-like receptor 4 (TLR4). In addition, the hippocampus is the center of the learning of associations between the environmental context and unconditioned stimuli in mammals (Kim and Fanselow, 1992; Bohbot and Corkin, 2007). Based on these studies, it is conceivable that TLR4 signaling within the hippocampus may play a crucial role in modulating memory of the rewarding effects of cocaine (Correia et al., 2020). This hypothesis was identical to our results of the vehicle-treated mice that developed a special preference for cocaine-paired context always displayed higher expression levels of TLR4 than in saline group animals. Indeed, a previous study determined that the TLR4 activation within the striatum evoked by repeated exposure to cocaine led to addictive behaviors (Northcutt et al., 2015). Additionally, these data also suggested that administration of the anti-inflammatory agent with the ability to suppress TLR4 during the destabilization period (known as extinction) was presumed to be effective at weakening the strength of cocaine-associated memory, facilitating extinction, and blocking cocaine-mediated CPP reinstatement *via* TLR4 signaling suppression.

### Potential Mechanisms of GLP-1R Agonist Ex4’s Behavioral Actions

Indeed, previous evidence has shown that the GLP-1 receptor agonist Ex4, a known neuroprotective agent (Mandal et al., 2018), suppresses TLR4 signaling (Ajay et al., 2011), and modulates memory formation (Oka et al., 1999), which are heavily involved in cocaine addiction (Hyman et al., 2006). Moreover, the specific TLR4 antagonist naltrexone has been effective at preventing the development of cocaine place preference in rats (Northcutt et al., 2015). Hence, it is conceivable that Ex4, which can attenuate TLR4 signaling in the hippocampus, may profoundly suppress the original cocaine-induced CPP memory and produce the new learning process, thereby enhancing the extinction of cocaine-induced CPP and reducing the CPP reinstatement. Importantly, this hypothesis has been validated by Ex4-treated mice with lower CPP scores. This is in line with a previous study indicating that memory retention could be disrupted after post-learning manipulations (Grecksch and Matthies, 1980). The expression levels of TLR4 within the hippocampus reactivated by cocaine were clearly inhibited by Ex4, similar to a previous study showing that Ex4 plays an anti-inflammatory role (Ajay et al., 2011; Zhu et al., 2021). Consequently, the GLP-1R agonist Ex4 is capable of modulating cocaine-related CPP memory, enhancing extinction, and weakening the cocaine-primed reinstatement by suppressing TLR4 over-expression within the hippocampus, similar to many studies revealing that extinction in substance abuse can be pharmacologically enhanced (Botreau et al., 2006; Paolone et al., 2009).

Although we have demonstrated that Ex4 facilitates the extinction of cocaine-induced condition place preference *via* inhibiting TLR4 signaling in the hippocampus, it is essential to explore the possible direct targets of Ex4. For instance, a previous study has demonstrated that peripheral administration of Ex4 inhibits the neurotransmitter dopamine release in the nucleus accumbens and the dorsal striatum as well as Lateral septum (LS) after cocaine exposure (Egecioglu et al., 2013; Sorensen et al., 2015; Reddy et al., 2016). More importantly, these inhibitory effects of GLP-1 receptor agonists on cocaine-induced behaviors may be linked to the inhibition of dopamine D1 receptor signaling in the ventral striatum (Sorensen et al., 2015). Accordingly, we could speculate that the suppressive effects of Ex4 on cocaine-mediated behaviors might be strongly related to other regions of the brain and inhibition of the dopamine release. In the future study, we focus on the effects of direct injection of Ex4 into the mesolimbic reward system such as the accumbens core and shell, Ventral tegmental area (VTA), and Lateral septum (LS) on the dopamine release and the potential mechanism. Meanwhile, there was a range of additional limitations and questions in this study that cannot be overlooked. First, the reason why Ex4 failed to modulate the locomotion of cocaine-induced reinstatement was not involved in this study. Furthermore, only CPP, one animal model of psychostimulatory drugs of abuse, was involved in the present study. Therefore, further studies are needed to elucidate the role of Ex4 in other animal models of drug addiction. It is also poorly understood whether other factors including stressful events following cocaine exposure participate in regulating cocaine-related maladaptive behavior. Additionally, the role of sex differences in the efficacy of GLP-1 receptor agonists in reducing cocaine-mediated behaviors has not been examined. Undeniably, further research is urgently needed to address these scientific questions.

In conclusion, the current research indicates that Ex4 administrated during the 6 h critical time window immediately after daily extinction training facilitates the extinction of cocaine-induced CPP and inhibits the expression levels of TLR4 signaling in cocaine-experienced mice. Considering that the Ex4 treatment ameliorates the maladaptive behavior of cocaine, Glucagon-like peptide-1 agonists might be repurposed as an effective medication that can reduce the risks of relapse of cocaine use disorder.

## Data Availability Statement

The original contributions presented in the study are included in the article, further inquiries can be directed to the corresponding author/s.

## Ethics Statement

The animal study was reviewed and approved by Ningxia Key Laboratory of Cerebrocranial Disease and Animal Research Ethics Committee of Ningxia Medical University.

## Author Contributions

All authors participated in the study design, data collection, analysis of data, and preparation of the manuscript. TH, TS, and FW participated in the designation of the experiments, preparation of the manuscript, analysis of data, and in the revision. CZ designed the experiments, performed the experiments, analyzed data, made the figures, performed the statistical analysis, and drafted the manuscript. All authors read and approved the final manuscript, and take responsibility for the integrity of the data and the accuracy of the data analysis. All authors contributed to the article and approved the submitted version.

## Conflict of Interest

The authors declare that the research was conducted in the absence of any commercial or financial relationships that could be construed as a potential conflict of interest.

## Publisher’s Note

All claims expressed in this article are solely those of the authors and do not necessarily represent those of their affiliated organizations, or those of the publisher, the editors and the reviewers. Any product that may be evaluated in this article, or claim that may be made by its manufacturer, is not guaranteed or endorsed by the publisher.
